# Supplementary material for: Are carbon emissions trading and green financial instruments synergistic? -Comprehensive quantitative research based on content analysis
Source: PLoS One. 2024 Mar 7;19(3):e0298601. doi: 10.1371/journal.pone.0298601 (PMC10919601; doi:10.1371/journal.pone.0298601)
Supplement: S1 Data — (ZIP) [file pone.0298601.s001.zip › Data/地级市Z_F工作报告.zip!/░▓╗╒/░÷▓║╩╨/└┤╫╘╣1⁄2╓┌║┼í╛┬φ┐╦╩2╛▌═°í┐_2016.docx]

各位代表：

现在，我代表市人民政府向大会报告工作，请与《蚌埠市国民经济和社会发展第十三个五年规划纲要（草案）》一并审议，并请市政协委员和其他列席人员提出意见。

一、努力攻坚，圆满收官“十二五”

2015年，全市各族人民在市委的坚强领导下，认真贯彻党的十八大、十八届三中、四中、五中全会和习近平总书记系列重要讲话精神，以“适应新常态、掌握新要求、抢抓新机遇、主动新作为、实现新发展”大讨论活动为开篇，统筹推进稳增长、促改革、调结构、惠民生等重点工作，各项主要经济指标稳中有升、升中趋优，社会大局和谐稳定，较好地完成了市十五届人大三次会议确定的各项目标任务。预计，全市实现地区生产总值1280亿元，同比增长10.5%左右，连续18个季度高于全省，其中一、二、三产增加值分别增长4.2%、11.5%、12%。财政收入228亿元，增长9.5%左右；其中地方收入119亿元，增长13.4%。固定资产投资1468亿元，增长18%。社会消费品零售总额570亿元，增长12.7%。城乡居民人均可支配收入26368元、11625元，增长9.2%、10.6%，分别居全省第2和第1。居民消费价格涨幅低于省控目标，万元地区生产总值综合能耗、主要污染物排放量持续下降。

（一）突出扶持实体经济，发展势头持续巩固

服务机制不断健全。出台促进经济持续健康发展18条等扶持政策，统筹安排各类发展资金38亿元，减免抵退企业各项税费39.5亿元，千方百计帮企业、助发展。新增贷款182亿元，增长18%。设立5亿元中小企业续贷转贷过桥资金，开展“税融通”业务和“金融服务进百企”活动。新增新三板挂牌企业10家。发放商标权、专利权质押贷款3.1亿元。全面推行“三证合一”、“一照一码”工商登记模式，新增各类企业6500户、认缴资本450亿元。

项目投资提质增效。完成“3461”项目投资830亿元，在建亿元以上项目320个。方兴科技柔性镀膜、MEMS产业化基地一期等140个项目建成投产，铜铟镓硒薄膜太阳能电池、中港IC产业园等200个项目开工建设。抢抓国家补助重大项目资本金政策机遇，争取城区老工业区搬迁改造等专项建设基金60.7亿元，居全省前列。成功开展“台湾招商周”、“粤闽招商周”等系列招商活动，积极参与省资本要素对接会、中博会等区域合作交流，豪威科技交互式电子白板、国显柔性OLED显示模组等项目顺利落户。新签约中电科41所现代通信仪器与自动化设备研发及产业化、与环球医疗合作共建医疗机构开发健康养老产业等30个央企合作项目，总投资528.1亿元。引进市外亿元以上项目资金770亿元，实际利用外商直接投资12.5亿美元，均增长10%。

工业经济企稳向好。握紧拳头、决战工业，出台聚焦工业突破政策，规模以上工业企业超过1000户，实现增加值670亿元、居全省第4，增长11%。工业经济效益综合指数居全省第2。德豪润达倒装芯片、大富工业机器人等29个项目建成投产，中创钛酸钡、心里程智慧园、自行车产业园等40个项目开工建设。获批省级硅基新材料战略性新兴产业集聚发展基地，设立30亿元硅基新材料产业发展基金。参与设立23亿元省高新投叁号基金，新增国家高新技术企业37家，实现高新技术产业产值920亿元、增长20%。玻璃设计院入选国家国际科技合作基地。“WM众创智慧谷”纳入国家级科技企业孵化器管理支持体系。发明专利授权量660件，增长12%。老工业区搬迁改造获省批准，城市西部化工企业退市进园全面启动，丰原集团明胶一期、环酸项目等基本建成。建成中小企业标准化厂房35.7万平方米，入驻孵化企业144家。成功举办安徽省第二届江淮杯工业设计大赛。

服务业持续繁荣。获批全省首家全国电子商务与物流快递协同发展试点城市，蚌山区入选省级电子商务示范区，经开区、蚌山区电商产业园和怀远县电商孵化园投入运营。徽商物流园、普洛斯仓储等项目加快推进，杭州费舍尔、高远物流等知名物流企业成功落户。中通、圆通总部项目入驻我市，实现快递业务收入2.8亿元、增长90%，分别居全省第3、第1。鹏欣水游城、金大地银泰城等项目进展加快，万达二期、百大名品中心建成运营。净增限额以上商贸企业40户、规上服务业企业10户。蚌埠（皖北）保税物流中心封关运营，当年业务量突破1亿美元，并成功引入首家跨境电商服务平台。实现进出口总额22.9亿美元，增长10.1%。花鼓灯嘉年华夜游项目、谷阳城遗址公园一期建成开放，旅游总收入增长15%。

农业发展有效提升。深入推进粮食生产三大行动和粮食高产创建活动，粮食总产286.6万吨，实现“十二连丰”，增长4.02%、居全省第2。五河现代牧业液态奶二期项目竣工投产，固镇亿只肉鸡项目新增养殖规模4000万只，新增怀远百万头美味猪现代养殖等3个种养加一体化项目。创建省级现代农业示范区1个、产业化示范区2个，新建市级现代农业示范区10个。土地流转面积达206万亩，流转率49%。新增有效灌溉面积9万亩。建设高标准基本农田29.5万亩，连续17年实现耕地占补平衡。农村土地承包经营权确权登记颁证全面推进，农村“两权”抵押贷款实现县域全覆盖。完成粮安工程危仓老库维修改造65万吨。全省春季农业生产现场会在我市召开，粮食绿色增产“蚌埠模式”在全省推广。成功承办第六届中国苜蓿发展大会暨国际苜蓿会议。

（二）着力强化城乡建设，城市功能日趋完善

城市建设加快推进。围绕“三环六轴三带”持续推进城市大建设，实施项目181个、完成投资248.6亿元，征迁312.9万平方米，拆违49.6万平方米，交付净地15975亩。中环线秦集、城南等路段全线完工，长淮卫淮河公路桥及北接线工程进展顺利。国道206、104和省道306、307境内部分路段提升改造工程顺利实施，全省首个运用PPP模式且投资额最大的交通基础设施项目—蚌五高速公路开工建设。新启动柴油机厂B区、蒋岗巷等41个棚户区项目，回迁安置16128户，货币化安置5948户。开工建设保障性安居工程60200套，基本建成13900套，公租房实物配租10726套。争取省代发地方政府债券37.4亿元。新设兴蚌一、二、三号城市发展基金，规模65亿元。博物馆、规划馆、档案馆建成开放。

文明创建力度加大。成功创建国家园林城市，并跻身全国文明城市提名城市，启动实施“九城同创”。集中整治老旧小区、背街小巷、农贸市场等重点区域环境。持续强化三车整治，联合治理非法营运三轮车。着力推进户外广告整治，加强店招店牌管理，建成延安路、解放路、东海大道等15条示范路。市区环卫保洁机械化水平显著提升，市容管理巡查体系框架初步搭建。不文明祭祀行为集中整治效果明显。城市形象宣传片《印象蚌埠》成功首发。

生态建设成效显著。淮河南岸滩地环境综合整治、天河—张公湖连接等工程加快实施，全国水生态文明城市建设试点工作有序推进，成功获批国家生态文明先行示范区。新增、改造绿地355.52万平方米，新增街头游园11个，建设绿道20公里，成片造林8.2万亩，建成森林长廊示范段167公里。积极开展历史遗留工矿废弃地复垦利用试点。扎实开展秸秆禁烧工作，实现卫星监测全年全域零火点目标。深入实施大气污染防治百日行动，PM10平均浓度下降到90 微克/ m3，降幅达22%。20个美好乡村建设重点示范村按时建成。

（三）持续保障改善民生，社会大局和谐稳定

民生实事深入实施。投入资金50亿元，按时保量完成33项省级民生工程。城乡居民医保财政补助标准由320元提高至380元。保障城乡低保160万人次、发放低保金3.8亿元。资助困难家庭学前儿童和贫困大学生171万元。维修改造校舍9.2万平方米。为8.8万名老年人发放高龄津贴3725万元。为3.9万名老人发放居家养老服务补贴2171万元，新增养老服务机构床位2080张。解决29.86万农村居民饮水不安全问题。发放特殊人群免费乘车补贴1130万元。奖补新建设施蔬菜基地800万元。投资6956万元，对7080户农村危房实施改造。新建、改造公厕32座，完成25个街巷小区路灯改造。实施精准扶贫、精准脱贫，减少贫困人口2.18万人。

保障体系逐步健全。新增城镇就业8.2万人，居全省第3。企业退休职工养老金月人均增加200元，连续11年提高标准。城乡居民基础养老金月发放标准提高至80元，惠及城乡居民42万余人。大病医疗救助金支付限额由15万元提高至30万元，贫困残疾人康复救助超额完成。住房公积金缴存率达77%，居全省第1；累计发放贷款79.3亿元，存贷比达95.3%、居全省第3。

社会事业协调发展。新建、扩建幼儿园55个，组建教育集团25个，在省内率先实现国家义务教育发展基本均衡县（区）全覆盖。安徽科技学院蚌埠校区、蚌埠职教园区、安财商学院开工建设。市中医院新院区、市疾控中心建成投用，市一院病房大楼、蚌医二附院新院区开工建设。在全国率先开展药品集中带量采购，药价平均降幅达19%以上。完善人才特区建设1+X政策体系，10人入选首批省特支计划，省“115”产业创新团队达15家。建成市少儿图书馆、美术馆，晋升国家一级文化馆2个。花鼓灯舞蹈《小小传承人》荣获全国少儿舞蹈“小荷之星”金奖，《玩灯人的娃娃们》获首届亚洲国际青少年微电影节金奖。青少年校园足球“百千万”工程全面实施。“石榴娃守护工程”获第二届中国青年志愿服务项目大赛金奖。全民国防教育更加深入，国防后备力量建设扎实有效。全国双拥模范城市“六连冠”创建工作务实开展。民族宗教、外事侨务、对台等工作不断加强，老年人、残疾人、妇女儿童、青少年等事业持续推进，消防、防空防震、应急等减灾防灾体系更加健全，统计、气象、档案、地方志、社会科学、文学艺术等服务经济社会发展能力进一步增强。

社会治理不断加强。开展信访积案化解攻坚活动，信访形势总体平稳。实施安全生产“铸安”行动，创新建立问题清单制度，强化危化品、消防、电梯等领域隐患排查整治，安全生产形势保持稳定。荣获全省首批无传销城市称号。建立重大不稳定问题清单制度，强化社会稳定风险评估工作。深入推进“四项建设”、“守护平安”系列行动，严厉打击各类违法犯罪，强化食品药品安全监管，市民群众安全感、满意度持续提升。

过去的一年，我们切实加强自身建设，巩固党的群众路线教育实践活动成果，扎实开展“三严三实”专题教育，政府系统工作作风得到进一步改进。坚持简政不简单、放权非放任理念，大力推进“放管服”改革，在全省率先公布市级政府权力清单、责任清单和涉企收费清单，创新出台政府权力运行监督管理实施办法，着力强化部门间的协作和上下级的衔接。完成新一轮政府机构改革，将15个职能相近部门整合为7个，改革力度全省最大。全面启动不动产统一登记改革工作，市县不动产登记工作机构、职能在全省率先整合到位。获批省唯一公共资源交易领域相对集中行政处罚权试点市。公共信息化平台建设全面启动。公务用车制度改革基本完成。全市“三公”经费下降21.8%。自觉接受人大法律监督和政协民主监督，办理市人大代表议案9件、建议148件，市政协提案56件。2014年度目标管理绩效考核居全省第4，连续三年跻身全省前5。推进依法行政，强化监察和审计工作，严肃查处了一批违纪违法案件。

各位代表！

2015年，是蚌埠经济社会发展取得显著成效的一年，实现了“十二五”圆满收官。这是多年积累的释放，也是多年心血汗水的结晶。“十二五”，我们品尝了艰辛，经历了难忘，收获了自豪。

五年奋斗，经济总量翻一番，综合实力大幅提升。地区生产总值年均增长11.7%，总量实现翻番、位次前移2位，人均生产总值超过6000美元。财政收入、地方收入分别是2010年的2.3倍、2.8倍。累计完成固定资产投资5000亿元，年均增长20%以上。京沪高铁蚌埠南站投入使用，合蚌客专、蚌淮高速建成通车，蚌埠新港开启通江达海新时代，综合交通枢纽地位更加巩固。新增国家农业科技园区、大禹文化产业示范园区等国家级战略平台，经济增长的平衡性、包容性、可持续性逐步增强。

五年聚焦，产业结构更优化，发展方式加快转变。二三产业比重达到85.3%，提高4.3个百分点。蚌埠国电、德豪润达、大富机电、中科电力等重大工业项目建成投产，规上工业增加值年均增长16.2%、总量前移6位，工业化率提高4.1个百分点。国家高新技术企业达204家，高新技术产业增加值占规上工业比重达38%，战略性新兴产业产值年均增长35.8%。义乌国际商贸城、万达广场、花鼓灯嘉年华、国际汽车城等一批现代服务业项目投入运营，区域商贸重镇重塑步伐加快。

五年攻坚，城乡建设快推进，承载功能显著增强。城市总体规划（2012-2030）成功获批，部分区划调整顺利完成，市区面积由601.5平方公里扩大至956.9平方公里，市区建成区面积达138平方公里、常住人口130万人。累计完成城市大建设投资1008.7亿元，大庆路淮河公路桥、市民广场等重大基础设施建成投用。改造棚户区（城中村）169个，62410户棚改居民实现“安居梦”。深入实施千万亩森林增长工程、绿满珠城行动，成片造林47.5万亩、是“十一五”的近6倍，新增、改造绿地924万平方米。怀远“皖北最美县城”建设加快推进，五河“水韵独具最美皖北水乡”日益彰显，“大美固镇”旧貌换新颜，市县联动发展格局逐步形成，城镇化率达52%。

五年开拓，改革开放再突破，发展活力不断迸发。投融资、医药卫生、文化体制等改革稳步推进，行政审批、“三个清单”、招投标管理、便民商贸流通体系等改革走在全省前列，棚改“蚌埠模式”、医患纠纷第三方调处机制等经验在全国、全省推广。民营经济比重提高8个百分点。新增蚌埠（皖北）保税物流中心、铁路无水港等平台，累计实现进出口总额80.8亿美元，是“十一五”的4.2倍。玻璃设计院、丰原集团等“走出去”步伐加快。招商引资成效显著，累计引进市外亿元以上项目资金2500亿元，是“十一五”的2.2倍。

五年倾力，社会民生新加强，百姓福祉持续改善。城乡居民收入年均分别增长11.4%、15.87%。新增就业32.69万人。创新建立老龄津贴、灵活就业人员生育保险等制度，城乡低保、社会保险待遇、最低工资等全部提标扩面，医疗和养老保险制度、就业服务网络等实现城乡全覆盖。教育事业加快发展，文化软实力不断提升，城乡公共卫生体系逐步完善。2527户以船为家渔民在全省率先上岸安居。新增环保公交车918辆，新增、优化公交线路41条，龙子湖、张公山等公园焕然一新，新建、改造公厕190座，人均住宅建筑面积由29.3平方米增加到38.6平方米，入选全国百个宜居城市。

各位代表！

五年的发展成就令人鼓舞，五年的奋斗进程历历在目。五年来，我们不畏艰险，共同应对严峻复杂的宏观形势，在经济新常态中展现了发展新姿态。五年来，我们携手并肩，协作共进，形成了全市上下齐心协力谋发展、奋发向上创一流的良好局面。今天的蚌埠，重返全省第一方阵其势已成、其时亦至。

各位代表！

今天的成绩，是省委、省政府和市委正确领导的结果，是全市上下团结奋斗、顽强拼搏的结果，是几大班子历届历任老同志打下坚实基础的结果。在此，我谨代表市人民政府，向全市人民，向人大代表、政协委员，向离退休老同志，向各民主党派、工商联、无党派人士，向各人民团体，向驻蚌解放军、武警官兵和政法干警，向国家和省驻蚌单位，向所有关心和支持蚌埠发展的各界朋友，表示衷心的感谢和崇高的敬意！

我们也清醒地认识到，前进道路上还存在一些困难和问题。主要是: 经济总量仍不大，牵动性强的大企业、大项目、大品牌仍不多，新的经济增长点还没有形成规模。工业经济主导地位不突出，整机、整器、整车类产业链末端、价值链终端产品缺乏，重点产业聚集度有待提升，开发区支撑作用仍需加强。重塑区域商贸重镇还有较大空间，现代服务业尤其是生产性服务业、新兴服务业发展仍然不够，文化资源没有得到充分挖掘。农业大而不强，农村基础设施还有比较薄弱的地方，县域经济、镇村经济发展滞缓，统筹城乡发展的任务仍然艰巨。各区功能定位和差异化发展不够明晰，教育、医疗等公共服务与中心城市地位相比还有一定的差距。老旧小区综合环境还没得到根本改善，物业管理、停车设施等公共服务保障能力距群众需求还有较大差距。大气污染防治尤其是雾霾治理形势不容乐观，环境保护和生态建设任务还十分繁重。城乡居民收入持续增长支撑和动力有限，涉及广大群众切身利益的就近就学就医就业问题还没有彻底解决好，养老服务、健身休闲、便民消费等发展水平不能有效满足群众需求。城市品质和市民品格仍需持续提升和持久养成。政府职能转变还不完全到位，一些部门思想解放不够、执行落实不力等“不严不实”问题依然存在，腐败现象还时有发生。对这些问题，我们将保持清醒头脑，采取切实有效措施，破解瓶颈制约，补齐发展短板。

二、奋力冲刺，赶超决胜“十三五”

“十三五”是我市全面建成小康社会的决胜时期，也是全面冲刺第一方阵的跨越阶段。尽管我们还会遇到一些困难和问题、风险与挑战，但综合判断，经济长期向好的基本面没有改变，仍将处于大有作为的重要战略机遇期。中央明确指出，2016年和今后一个时期，要在适度扩大总需求的同时，着力加强供给侧结构性改革，实施宏观政策要稳、产业政策要准、微观政策要活、改革政策要实、社会政策要托底的五大政策支柱，明确诸多新论断、新政策、新举措，传递出一系列含金量十足的政策导向。省委、省政府在“十三五”规划中明确提出要推动淮河流域综合治理和振兴发展，并以此为加快皖北发展的主要抓手，推动淮河生态经济带建设，围绕综合交通、航道整治、港口建设、产业振兴等领域谋划和实施一批有带动力的重大项目，合芜蚌作为安徽省依托城市被纳入国家全面创新改革试验区，各种政策叠加效应正在集中释放。同时，经过多年的努力，我市综合实力大幅提升，经济转型升级成效初显，争先进位、跨越发展的势头日趋强劲。更难能可贵的是，干部队伍的观念理念、思想思路和精神状态加速转变、有效提升，广大市民热爱蚌埠、发展蚌埠、奉献蚌埠的激情不断释放。我们坚信，只要全市上下凝心聚力、团结奋进，持续不断地破解发展难题、增强发展动力、厚植发展优势，就一定能够谱写蚌埠加速崛起、赶超跨越的新篇章！

市委十届十八次全会通过的《中共蚌埠市委关于制定蚌埠市国民经济和社会发展第十三个五年规划的建议》明确，“十三五”时期发展的指导思想是：高举中国特色社会主义伟大旗帜，全面贯彻党的十八大和十八届三中、四中、五中全会精神，以马克思列宁主义、毛泽东思想、邓小平理论、“三个代表”重要思想、科学发展观为指导，深入贯彻习近平总书记系列重要讲话精神，认真落实“四个全面”战略布局，坚持发展是第一要务，牢固树立创新、协调、绿色、开放、共享的发展理念，紧紧围绕“全面建成小康社会、全面冲刺全省第一方阵”目标，以提高发展质量和效益为中心，以加快调结构转方式促升级为主引擎和总抓手，以增进人民福祉、促进人的全面发展为出发点和落脚点，大力推进新型工业化、信息化、城镇化和农业现代化，加快建设宜业宜居宜游的皖北中心城市。

在市委《建议》的指导下，市政府编制了《蚌埠市国民经济和社会发展第十三个五年规划纲要（草案）》，提出今后五年经济社会发展主要奋斗目标是：地区生产总值年均增长9.5%，经济总量力争较2015年翻一番，进入全省第一方阵；人均生产总值达1万美元以上，力争达到全国平均水平；财政收入增长9%以上；固定资产投资增长11%；进出口总额增长高于全省水平；城乡居民人均可支配收入分别达到41500元、18300元。主要污染物排放得到有效控制，资源利用效率显著提高，生态环境质量明显改善。

今后五年的主要任务是：

（一）坚持创新发展，加快经济转型升级步伐。始终把创新摆在发展全局的核心位置，系统推进国家全面创新改革试验和创新型城市建设，突出抓好重点领域和关键环节改革，充分发挥市场在资源配置中的决定性作用，加快形成有利于科学发展、转型升级的体制机制。深入实施调转促“486”行动计划，加快构建更具竞争力的现代产业体系，二三产业比重突破90%。牢固树立“发展经济，工业优先”的理念，着力培育大产业、构筑大园区、引进大企业、建设大项目、打造大品牌，努力打造工业经济升级版。规模以上工业企业突破2000户，规上工业增加值年均增长11%，工业化率超过50%。加速实施战略性新兴产业“四个一行动计划”，高新技术产业、战略性新兴产业增加值占规模以上工业比重分别超过50%、45%，形成硅基新材料、电子信息、装备制造3条千亿产业链。以扩大服务消费为重点带动消费升级，推动生产性服务业向专业化和价值链高端延伸，生活性服务业向精细化和高品质转变。现代服务业增加值占服务业比重达60%以上，生产性服务业占服务业比重达50%。加快粮经饲统筹、农林牧渔结合、种养加一体、一二三产融合发展，期末农产品加工产值达到1500亿元，比2015年翻一番。注入绿色低碳技术，融入文化创意元素，引入农业科技成果，深度保护和科学利用农业生态资源，大力发展现代生态农业。实施“互联网+”蚌埠行动计划，拓展网络经济空间，推动新技术、新产业、新业态蓬勃发展。实施产业品牌提升工程，加速挖掘、生成一批整机、整器、整车类价值链末端、产业链终端产品，推动“蚌埠品牌”向“安徽品牌”、“中国品牌”升级。2018年底前完成西部城区化工企业异地技改、整体搬迁，搬活企业、搬强产业、搬美城市、搬乐百姓，改造、再造一个产城一体的现代化西部新城区！

（二）坚持协调发展，着力形成统筹推进格局。坚持“全域蚌埠、多规融合”，全面实施“三环六轴三带”城市发展框架，推进滨湖新区、高铁新区、城南新区、淮上新区“四大板块”建设，加强蚌（埠）淮（南）同城化发展，打造皖北地区装备制造、综合交通、科技教育、医疗卫生、商贸物流、文化旅游、金融服务中心。期末，市区建成区面积达到165平方公里，常住人口达到165万人。坚持重大产业项目向县域布局、重大基础设施向县域延伸、优质资源要素向县域集聚，不断增强县域经济发展活力和内生动力。三县县城建成区面积均超过20平方公里，县域经济总量比重达50%以上。扎实推进以人为核心的新型城镇化，促进有能力在城镇稳定就业和生活的农业转移人口举家进城落户。全面实施美丽乡镇建设、中心村建设和自然村环境整治，实现美丽乡村建设由“以点为主”向“由点带面”的战略转换。大力推进“军转民”、“民参军”，推动军民深度融合发展。依托国家级大禹文化产业示范园区，加快构建门类众多、特色鲜明的现代文化产业体系，延续城市历史文脉。文化产业增加值年均增长25%以上，占地区生产总值比重达8%以上。坚持深入推进全国文明城市创建工作，持续提升城市品质和市民品格，涵养新时期“蚌埠精神”，让每一位在家的、走外的，本地的、新来的这方水土上的人们，都能更加自豪地说：我是蚌埠人！

（三）坚持绿色发展，努力营造美丽生态家园。坚定走生产发展、生活富裕、生态良好的文明发展道路，把好山好水好风光融入城市。统筹荆山、涂山、天河生态规划，倾力打造“荆涂淮河湾”。加快淮河、浍河、怀洪新河等13条河道和龙子湖、沱湖、四方湖等9个湖泊水环境综合整治，加强天河、四方湖、芡河等饮用水源地保护、利用，建成国家级沱湖湿地自然保护区、三汊河湿地公园，构建“河畅、水清、岸绿、景美”的水生态体系，让灵动之水滋润魅力珠城。大力实施生态造林和绿化工程，森林覆盖率突破20%，人均公园绿地面积达到13平方米以上，城市建城区绿化覆盖率达到44%以上，完善提升国家园林城市水平，争创国家森林城市。强化约束性指标管理，实行能源和水资源消耗、建设用地等总量和强度双控行动。推进多污染物综合防治和环境治理，实行秸秆禁烧等联防联控和流域共治，实施工业污染源全面达标排放计划，坚决打好大气、水、土壤污染防治“三大战役”。加大农村面源污染防治力度，统筹农村饮水安全、污水垃圾处理，推进种养业副产品资源化利用、无害化处理。健全完善生态文明制度体系，推进产业生态化、生态经济化，承担好国家生态文明先行示范区建设任务和示范责任，更加彰显水在城中、城在林中、城依山、水映城的山水园林城市！

（四）坚持开放发展，全面提升对外开放水平。全面对接、融入“一带一路”、京津冀协同发展、长江经济带“三大战略”，参与长三角区域分工与合作，坚持进口与出口并举、引进来与走出去并重、引资和引技引智齐抓，全面提升开放型经济水平。推进大通道大平台大通关建设，支持和引导保税物流中心提升服务功能，谋划建设综合保税区。大力实施口岸提升、主体培育、出口品牌和进口促进、市场多元化战略，全面提升口岸功能和贸易便利化水平，扩大进出口贸易规模，促进外贸增长方式转型升级。创新招商引资模式，大力推进精准招商、产业链招商、以商招商，累计利用市外亿元以上项目资金3500亿元、外商直接投资80亿美元。加快人才特区建设，打造京沪线人才集聚高地。推动开发区由速度数量型向质量特色型转变，主导产业产值占比达到70%以上，工业产值占全市工业总产值70%以上，打造市高新区、蚌埠工业园2个千亿级开发区，经济开发区进入国家级开发区行列。加快推进4C级民航新机场、水蚌线外迁、怀远至淮南黄疃窑大桥、城市轨道交通等重大基础设施建设，完善构建“公铁水空”立体综合交通体系，让走南闯北的驿站更加温馨舒适，让承东启西的节点更加便捷畅顺！

（五）坚持共享发展，决战决胜同步全面小康。“民之所呼，政之所向。”我们将按照人人参与、人人尽力、人人享有的要求，着力强化民生保障，让发展成果更多、更公平地惠及全体市民。坚持优先发展教育，加大教育集团化推进力度，在全省率先普及学前到高中阶段15年教育，推动职业教育产教融合、校企合作发展，促进农民工子女平等接受教育，鼓励和支持社会资本进入教育领域，让更多的孩子在家门口享受到公平优质的教育资源。实施健康蚌埠建设工程，合理配置公立医院资源，发展各类特色专科医院，引进北京、上海等地优质医疗资源合作共建高层次特色医院，健全市县乡村四级医疗服务网络，打造“城市一刻钟、农村半小时”医疗服务圈，努力实现小病不出社区、村，大病不出蚌埠城。鼓励支持农副产品平价商店、便利店、早餐店、家政服务点等居民生活必备的商业网点建设，提速升级“15分钟便民服务圈”。高质量承办好省十四届运动会，为全省人民奉献一届科技省运、文化省运、生态省运、温馨省运，推动竞技体育、群众体育和体育产业快速发展，打造运动之城、健康之城、幸福之城。加快推进棚户区和城中村改造，实施老旧小区改造提升工程，促进房地产稳定健康发展。加强村级公路提级改造，实现县域内相邻乡镇通达三级以上公路，符合规划的30户以上自然村全部通达水泥（沥青）路。坚持普惠性、保基本、均等化、可持续方向，全面提高就业和创业服务、社会保障、食品安全、环境保护等公共服务共建能力和共享水平。坚持精准扶贫、精准脱贫，组织实施“一二三”扶贫开发行动计划，实现一年贫困村出列、二年贫困户集中脱贫、三年巩固提高，确保一个贫困村、一个贫困户不掉队。健全民生投入稳定增长机制，实现民生改善与经济发展同步共进、幸福指数与发展指数同步提升，努力让更多城乡居民学有优教、住有宜居、乐有美景、病有良医、老有善养！

三、全力以赴，锐意进取开好局

2016年是“十三五”开局之年，也是蚌埠冲刺全省第一方阵的关键之年，做好今年的各项工作意义重大。今年全市经济社会发展的主要预期目标是：地区生产总值增长9.5%左右；一、二、三产分别增长4%、10.7%和11%；规模以上工业增加值增长11.5%；财政收入增长9%；固定资产投资增长12%；社会消费品零售总额增长12%；进出口总额增长5%；利用外商直接投资增长10%；城镇常住居民人均可支配收入与经济增长同步，农村常住居民人均可支配收入增长高于经济增长；城镇登记失业率、人口出生率、居民消费价格涨幅、单位生产总值能耗、国家要求削减的主要污染物排放均控制在省下达的目标以内。主要经济指标增速继续高于全省、领先皖北。

为此，我们将重点做好九个方面的工作：

（一）聚焦工业突破，铸造升级主引擎

做大产业。全面实施调转促“486”行动计划，着力打造现代产业新体系。加快省级硅基新材料战略性新兴产业集聚发展基地建设，推进国家新型显示产业集聚发展试点，加快实施铜铟镓硒薄膜太阳能电池、台玻电子级玻璃纤维等项目，开工建设8.5代液晶玻璃基板、豪威科技交互式电子白板等项目，竣工投产玻璃设计院触摸屏用高强保护玻璃、兴科LOW-E镀膜玻璃等项目，延伸薄膜太阳能电池、OLED等硅基产业链。统筹推进高端装备、生物医药等战略性新兴产业发展，加快市级智能装备、生物制造、特色电子元器件战略性新兴产业集聚发展基地建设，开工建设中粮生化燃料乙醇搬迁升级等项目，加快推进中科电力装备工业园、大富机电高端装备产业园、丰原发酵3万吨L-苹果酸等项目，竣工投产凯盛工程建材行业机器人、雪郎生物年产1万吨PBSA等项目。加快实施蚌埠卷烟厂异地技改、国电蚌埠电厂二期扩建等项目，综合运用新技术、新工艺、新设备、新材料改造升级传统产业。强力推进中粮生化、丰原集团、八一化工等重点化工企业退市进园，实现技改升级新项目全面开工建设。

做强企业。全面落实减税降费政策，清理各种不合理收费特别是垄断性中介收费，帮助企业降低制度性交易成本、税费负担、财务成本、物流成本等。实施领军企业培育工程，集中资源支持优质企业滚动发展、做大做强。高新区、淮上区新增10亿元以上企业各3户，三县各2户，龙子湖区、禹会区各1户。实施规模以上企业突破工程，健全企业入规目标管理和服务机制，净增规模以上企业130户。实施中小企业雏鹰工程，支持中小微企业入园发展，新增专精特新企业30户、两化融合示范企业25户。实施企业竞争力提升工程，推动企业管理规范化、资产证券化，鼓励参与国家、行业和地方标准制定，争创省著名商标15件、名牌产品5个。

做优园区。推动集聚发展，引导企业和项目向工业园区和工业走廊集中布局，省级以上开发区工业总产值占比提升到60%以上。坚持错位发展，推行产业发展负面清单制度，省级以上开发区主导产业产值占比提升2个百分点。支持蚌埠铜陵现代产业园加快发展，合作共建蚌埠铜陵五河（头铺）产业园。全面启动怀远龙亢经济开发区起步区基础设施建设。

（二）壮大服务业，激发转型新活力

推动电商物流快递协同发展。完善政策扶持体系，强化要素统筹整合，深入实施国家级电子商务与物流快递协同发展试点。提升经开区、蚌山区电商产业园运营水平，新建高新区电商产业园，加快县域电商产业园建设，入园企业超过100家。推进怀远县全国电子商务进农村综合示范。鼓励企业自建平台和利用第三方平台拓展市场，新增实体企业开展电子商务20家。调整优化物流业布局，加快推进皖北徽商物流港、新港物流园、皖北杂粮交易中心、普洛斯仓储等项目建设，打造西部工业物流、北部商贸和保税仓储物流、南部电商物流等物流产业集聚区。鼓励社会资本进入快递服务领域，吸引大型快递企业在蚌设立皖北总部或转运中心。快递业务量达3900万件、增长50%。

改造提升传统商贸服务业。建成义乌国际商贸城工业加工区、中华玉博园、海吉星二期，持续增强市场集聚辐射功能。加快推进金大地银泰城、喜来登酒店、南翔广场一期等项目，初步形成东海大道高端商贸集聚带。加快建设天洋国际大厦、二马路市场及周边区域改造等项目，开工建设汇金商业广场二期，持续完善淮河路商圈。净增限额以上商贸企业30户、规模以上服务业企业15户。继续推进“15分钟”便民生活服务圈建设，新增社区便利示范店10个、放心粮油直营店（经销点）25个、主食厨房直营店15个。

加快培育服务业新业态。推进蚌埠旅游集散中心、白乳泉综合提升等项目建设，部分开放淮河蚌埠闸湿地公园，旅游总收入增长20%以上。发挥安徽后青春工业设计研究院示范带动作用，加快工业设计产业园建设。深入实施全国第二批信息消费试点，加快4G网络在城区深度覆盖和农村广度覆盖。支持鼓励总部经济、服务外包、咨询中介等现代服务业发展，积极培育家政服务、健康养老等消费新热点。

（三）发展现代农业，着力提高质量效益

加快农业产业化步伐。分级落实粮食安全责任制，粮食总产达288万吨。新增市级以上蔬菜标准园15个、大水面健康生态养殖面积4万亩，建立循环农业生态示范区1个，创建现代生态农业产业化示范县，推进农产品生产全绿色。实施畜牧业升级计划，加快怀远美味猪、固镇肉羊等项目建设，每个县新增1个种养加、一二三产融合互动的农业产业化项目，推动农产品生产全链条。鼓励标准化基地和示范区产品申报绿色食品、有机食品认证，新增农产品“三品”认证20个，推动农产品生产全品牌。培育20个市级以上农业产业化联合体、10个以上龙头企业“甲级队”。建立完善新型职业农民培训、认定、扶持工作机制。大力发展专业化代耕代种、统防统治、农机合作社等社会化服务组织，新增市级以上农民专业合作社30家。50亩以上的规模经营面积占土地流转总面积达47%。创建市级现代农业示范区3个。

提高农业基础设施装备水平。加快推进淮干蚌浮段行洪区改造、怀洪新河水系洼地整治等重点水利工程，开展生态清洁型小流域建设，实施农村河沟塘综合整治。新增有效灌溉面积10万亩，建设旱涝保收田10万亩。加大现代农业综合开发力度，完成高标准基本农田建设20万亩。新建农田林网23万亩。加快农机农艺融合，主要农作物耕种收综合机械化率达到80%以上。扶持育繁推一体化种业发展，支持新型经营主体开展物联网应用示范。

深化农村综合改革。推进农村集体产权制度改革。完成农村土地承包经营权确权登记颁证工作，建成市级农村集体产权交易市场，开展县乡级建设试点。深入推进农民土地承包经营权、住房财产权抵押担保贷款试点，发挥怀远作为全国农房抵押贷款试点县的示范作用，让资源变资本、效益增收益。探索建立农业转移人口市民化引导基金，在三县开展农民进城入镇改革试点，加快农民工市民化。深化户籍制度改革，允许农业转移人口等非户籍人口在就业地落户，把公租房范围扩大到非户籍人口。

（四）强化全域统筹，提升新型城镇化水平

做优做大中心城区。持续推动征迁拆违和城市大建设，实施大建设项目180个、完成投资240亿元。完成征迁300万平方米、交付净地1.5万亩。开工建设各类保障性安居工程24377套，基本建成12506套。提高国开行贷款使用率，棚改货币化、商品房安置比例达50%以上，在全国老工业基地城市中率先完成集中连片棚户区改造任务。加速推进长淮卫淮河公路桥、中环线特步园区段建设，全面贯通城市中环线。完成国道104、206和省道306、307境内提升改造工程。加快天河科技园、长淮卫临港产业园、马城新区建设，启动实施延安路跨河通道、4C级民航新机场项目，积极开展怀远至淮南黄疃窑大桥等前期工作。开工建设110千伏光彩变、220千伏东海变等输变电工程。加快高铁站东公交停保场、马城公交首末站建设，新增、优化公交线路5条，消除通达盲区。

加快县域城镇建设。继续推进“三线三边”整治和城镇“三治三增三提升”行动，加快实施规划提升、整治、畅通、拆违、绿化提升、出口整治六项行动，提升城镇综合承载能力。加快重点镇建设，打造龙亢、马城、新马桥、沫河口、沱湖等一批特色工业强镇、农业大镇、休闲旅游旺镇。加快美丽乡村建设，集中开展为期两年的乡镇政府所在行政村中心村建设，同步在乡镇政府驻地建成区开展以治脏、治乱，加强基础设施建设和公共服务配套为主的“两治理一加强”工作。新建改造农村电网线路1777公里。

精心精细管理城市。坚持把“一尊重五统筹”要求贯穿城市发展全过程，推动城市持续健康发展。加强对城市的空间立体性、平面协调性、风貌整体性、文脉延续性等规划和管控。加快推进政府公共信息化平台建设，基本建成公共管理、公共服务、公共信息、公共事业、公共应急五大云数据方向的数据交换以及各类信息化应用项目，让“数据多跑路、群众少跑腿”，全力打造智慧城市。健全联防共治的公共安全治理机制，着力铸造安全城市。加快推进市区道路环卫保洁全机械化、标准化作业。强力推进道路交通安全集中整治行动，持续强化“三车”治理, 严厉打击各类非法营运，加强自行车道和步行道体系建设，规范城区交通秩序。提升流动摊点饮食群、农贸市场管理水平，开展物业管理示范小区创建。启动实施老旧小区改造提升工程，着力推进老旧小区管理规范化、标准化、市场化。

（五）扩大有效投入，持续增添发展后劲

加快重点项目建设。严格项目工作责任制，落实“四督四保”调度机制，促进投资稳定增长和结构优化。建成投产LOW-E节能玻璃、德豪光电LED等项目，加速实施蚌五高速公路等项目，开工建设中粮粮食产业园一期、水蚌线外迁等项目，完成“3461”重点项目投资950亿元。精心筹备承办省十四届运动会，加快体育中心、体育场升级改造等场馆建设。细化、实化项目前期工作，加速实施棚户区改造、化工企业搬迁、市民政园等国家资本金项目建设。新增储备项目规模3000亿元。

提升招商引资实效。扎实开展“产业招商深化年”活动，围绕重点产业、主导产业开展产业链招商、园区招商、以商招商，提高招商引资精准度、产业布局集中度。每个县区至少引进20亿元以上产业类项目2个。紧盯“一线四点”（京沪高铁沿线、长三角、珠三角、环渤海、台湾）重点区域，招大引强、招高引新、招群引链、招才引智，引进新知科技产业园、芯华微波集成电路等一批大企业、大项目，实际利用市外亿元以上项目资金800亿元。积极参加省民企外企对接等活动，逐一推动与央企合作和资本要素对接项目的落地开工。创新招商引资政策体系，充分发挥产业基金、风险基金的引导促进作用，更多依靠市场的手段和优质的环境配置资源、引进企业。

不断拓宽融资渠道。健全以扶持实体经济效益、效果为核心的财政资金以存促贷机制，疏通金融进入实体经济的管道，新增贷款200亿元，增量存贷比力争达到100%以上。推动双环电子、佳先股份在创业板上市，新增10家新三板挂牌企业。积极争取省政府债券支持，发行蚌投公司债券和城投三期企业债券。整合资源，建立西部城区化工企业搬迁、旧址综合开发统一融资平台。筹集并运行首期规模30亿元硅基新材料产业发展基金。引进金融机构1家以上。完善金融监管体系和风险预警机制，依法严厉打击非法集资、非法吸储等涉众型经济犯罪，切实维护金融市场秩序。

（六）深化改革开放，加速释放发展新动力

着力攻坚重点领域改革。加强供给侧结构性改革，突出抓好去产能、去库存、降成本、补短板等关键任务，夯实经济稳定增长根基。深化商事制度改革，全面推进“三证合一、一照一码”，探索事中事后监管新模式。创新政府“三资”管理，推动混合所有制经济发展，民营经济比重提升1个百分点。探索实行国资分类管理、分类定则、分类考核，提高国有资本配置和运营效率。改进预算管理和控制，推进三年滚动预算管理。健全权责利相结合、借用还相统一的债务管理机制，实行总量控制、限额管理。做好建筑业、房地产业等领域营改增工作。深化医药卫生体制综合改革，强化“三医联动”推进公立医院建立管理运行新机制。提升基层医疗机构基础、环境建设和服务承载水平，初步建立分级诊疗制度。统筹推进职业教育、就业创业等领域综合改革，努力实现保障共享、服务均等，以群众获得感填写改革成绩单。

实施创新驱动发展战略。加快创新型城市建设，强化企业创新主体地位和主导作用，促进科技成果资本化、产业化，新增高新技术企业25家，培育省级以上研发平台10个。推进产学研用协同创新，重点支持平板显示、硅基新材料、电子信息等领域共性技术和关键技术研发，组织实施石墨烯电池产业化及其应用、第5代移动通信测试等重大科技攻关项目。加快高新区创客空间、经开区大学科技园等新型创新创业平台建设，全市在孵企业达300家。发明专利授权量730件。全面深化人才特区建设，力争引进高层次科技团队5个。

提升外向型经济水平。加强蚌埠（皖北）保税物流中心运营管理，推进与南京港合作建设公路无水港，申建进口农产品、水果和加工食品指定口岸。积极申报二类水运口岸，开展国际集装箱业务。依托铁路无水港申建一类铁路口岸，谋划开行中欧班列。继续支持重点骨干企业“走出去”，完成丰原集团巴西大豆和玉米深加工、匈牙利柠檬酸项目主体工程，加快实施玻璃设计院安哥拉浮法玻璃等项目。实现进出口总额和利用外商直接投资持续增长。

（七）推进文化立市，打造特色文化名城

大力弘扬先进文化。坚持以社会主义核心价值观为统领，持续弘扬“创新进取、责任担当、务实诚信、合作共赢”的先进思想文化。大力培育传承优良家风、校训、企业精神和乡贤文化，积极开展道德实践和志愿服务，广泛凝聚见贤思齐、崇德向善的力量。大力开展“书香满珠城”全民阅读等群众文化活动，支持“职工书屋”等建设。深入推进全国文明城市创建，广泛开展群众性精神文明创建活动，构建具有高度认同感和强大凝聚力的精神家园。

繁荣发展文化事业。加快实施公共文化服务设施“8633”工程，提标市级“三馆一院”、县级“两馆一场”，强化“工青妇科教”系统基层公共文化资源整合，提高各类文化设施的利用率。认真开展第一次全国可移动文物普查工作，加快推进双墩遗址申报国家考古公园和花鼓灯艺术申报世界非遗项目。组织实施舞台艺术精品工程、重大新闻出版工程、重大题材影视剧创作工程和文艺精品、优秀少儿作品创作工程，深度发掘蚌埠文化丰厚底蕴。办好“温馨蚌埠欢乐珠城”、“花鼓灯艺术交流展演周”活动，开展“文化惠民消费季”系列活动，持续活跃百姓大舞台、群众竞技场。

加快文化产业发展。健全落实督查、考核、奖惩等机制，全面提升大禹文化产业示范园区基础设施和服务功能。推动文化与科技、旅游、金融等深度融合，加快推进龙子湖环境综合治理核心区域、大明文化产业园、花鼓灯嘉年华二期等重点项目建设。大力发展文化创意设计、文化休闲娱乐、文化产品和设备制造、工艺美术品、互联网信息及广电影视服务等重点产业门类，打造印刷印务、专业化设计、玉器工艺品、影视制作、文化旅游等特色产业集聚地。

（八）坚持绿色发展，加强生态文明建设

加大生态保护和环境治理力度。启动创建国家生态园林城市、森林城市。强力推进大气污染防治工作，继续推行全年全域全面秸秆禁烧，持续治理建筑施工、道路扬尘、餐饮油烟，淘汰黄标车、老旧车4000辆，PM10浓度下降到85微克/m3以下。加强工业污染防治、农村畜禽养殖等面源污染治理。深入开展水污染防治行动计划，加强跨界污染综合防治，推进淮河、芡河、天河等饮用水水源地安全保障工程。加快推进天河—张公湖连接工程项目，有效改善席家沟沿线区域生态环境。继续实施千万亩森林增长工程，成片造林6万亩。新增城镇园林绿化面积317万平方米，改造绿地225万平方米，新建街头绿地（游园）20个，建成城市绿道50公里。

推进资源节约集约利用。严格落实污染物排放总量和能耗消费总量“双控制”。划定城市发展边界线、永久基本农田保护红线、生态保护红线。积极开展资源综合利用产品申报认定，提升工业固体废弃物、废气废水、余热余压综合利用率。清理处置批而未供、闲置低效土地5000亩，提高节约集约用地水平。实施华润雪花、中航三鑫等工业锅炉和建材窑炉脱硫工程。建成市第二、第三和怀远涡北、五河污水处理厂二期工程，新建污水管网80公里，新增污水处理能力12.5万吨。

加强生态文明制度建设。加快国家生态文明先行示范区建设，完善评价、考核、奖惩机制，实施海绵城市建设等十大重点工程。探索建立生态补偿机制，实行生态文明一岗双责，实施最严格问责机制，对破坏生态文明建设的企业停产、车辆停运、工地停工、商贸停业、干部停职。开展万家企业节能低碳行动、节能减排全民行动，办好节能宣传周、低碳日等生态文明主题活动，传承和弘扬生态文化。

（九）持续改善民生，维护社会和谐稳定

着力保障基本民生。深入实施33项省级民生工程，重点抓好重度残疾人护理、农村道路畅通等新增民生工程。完善城乡一体化创业就业服务体系，促进高校毕业生、就业困难人员等重点群体就业，大规模开展职业技能培训，新增城镇就业6万人以上。实施全民参保登记计划，扩大社会保险覆盖面。推进城乡居民医疗保险制度整合和机关事业单位养老保险制度改革。健全城乡一体化社会救助体系，加大对城乡低保户、五保户、孤儿、重症患者等困难群体的救助力度，多形式、多渠道开展慈善救助活动。完善宗教教职人员生活补贴增长机制。新购置环保公交车200辆。新建、改造公厕34座。

打好脱贫攻坚战。积极开展贫困户建档立卡“回头看”，落实扶贫对象需求、目标时限、措施、责任等“四个清单”。按照“五个一批”的要求，大力推进产业脱贫、就业脱贫和金融扶贫、社会扶贫等“九大专项行动”。加大各级财政扶贫投入，统筹各类涉农资金向贫困村、贫困户倾斜。完善扶贫责任绩效考评机制，全面落实扶贫对象帮扶到村、到户举措，确保88个贫困村全部出列，减少贫困人口3.5万人以上。

统筹发展各项事业。健全集团化办学机制，加快教育信息化建设，推动学前教育、义务教育优质均衡发展。深化职业教育综合改革，加快推进职教园区建设。实施乡村教师支持计划，加强乡村教师队伍建设。大力开展全民健身运动，推进校园足球“百千万”工程。提升计划生育服务能力，落实全面二孩政策。开展农村妇女“两癌”筛查工作。加快实施市一院病房大楼、市二院新院区、蚌医二附院新院区、市三院改扩建及妇儿病区等项目。加快构建气象现代化体系，全面提升气象综合服务能力。加强国防动员、国防教育和国防后备力量建设，推进军地融合发展。争创全国双拥模范城“六连冠”。统筹抓好民族宗教、防空防震、应急管理、外事侨务、档案、地方志、统计、审计等工作，加快残疾人、妇女儿童、青少年、老龄等事业发展。

创新社会治理方式。启动“七五”普法工作，推进法治蚌埠建设。完善社会治安综合治理体制机制，加快社会治安视频监控系统建设，力争城区重点部位和场所无盲区。健全六级防控体系，全面推进“一村一警”。积极推进阳光信访、责任信访、法治信访，深入开展社会矛盾排查化解。突出抓好危化品、特种设备、油气管线等重点领域安全生产，强化食品药品安全监管，依法严厉打击各类刑事犯罪，坚决维护社会大局和谐稳定。

各位代表！

全面建成小康社会、全面冲刺第一方阵，对政府工作提出了更高要求。我们将始终坚持忠诚、干净、担当，切实加强自身建设，不断提升工作效能、效率、效果，尽心竭力为人民服务、对人民负责、让人民满意。

我们将坚持依法行政，努力建设权责法定的法治政府。牢固树立简政不简单、放权非放任理念，推进行政审批服务标准化、实施全流程监管，强化政府权力清单、责任清单动态调整和监督检查，持续深化“放管服”改革。完善依法科学民主决策体制机制，加强政策执行督查督导和责任追究。自觉接受市人大及其常委会法律监督、市政协及各人民团体民主监督和社会舆论监督，积极配合人大代表和政协委员开展好视察、调研、问询等工作。深化行政执法体制改革，持续推动执法重心和执法力量下移。加强行政复议、行政应诉工作，主动协助人大更好行使地方立法权，让政府工作始终走在法治轨道。

我们将始终牢记宗旨，努力建设情系百姓的亲民政府。把群众获得感、社会满意度作为政府工作的晴雨表、风向标，全力以赴解民忧，全心全意惠民生，做到重大决策依民而定、重点项目应民而立、重要工作为民而行。建立健全调查研究常态化工作机制，捧出真心问政于民、问计于民、问需于民。加大社会事业领域政府购买服务力度，加快文化、卫生等事业单位创新管理、分类改革。完善人大代表议案、建议和政协委员提案办理机制，加大网上办理、公示力度。推动市长热线、政风行风热线等平台功能再完善、服务再优化、效率再提升，事事汇聚真情，时时倾注民心。

我们将强化责任担当，努力建设勤政高效的务实政府。坚定自我革新的胸怀和责任，强化锐意攻坚的勇气和担当，用改革的办法、开放的思路和务实的举措，各项工作奋力“进五争三创一流”，增强可持续跨越发展能力。持续完善政策落实和工作推进机制，实行清单式管理全覆盖，让广大干部心中都有“时间表”，眼前都有“施工图”，肩上扛着“任务书”。深入推进治庸治懒，强化干部问绩问效，坚决整治慢作为、严惩乱作为、杜绝不作为。强化目标考核过程管理，优化评价指标体系，分层次分类别开展差异化考核，引导各县区错位发展。推动政府重大事项在更宽领域、更大范围公开，便捷信息共享、回应社会关切。

我们将推进从严治政，努力建设风清气正的廉洁政府。巩固“三严三实”专题教育成果，严守各项政治纪律和政治规矩，严格落实中央八项规定、省委“六个坚持”、“九个不要”等作风建设各项规定，持之以恒纠正“四风”。深入推进政府系统党风廉政建设，认真落实“一岗双责”，切实履行主体责任，旗帜鲜明地支持纪检监察机关履行执纪监督责任，推进审计监督等权力运行监督“四个全覆盖”。严格控制“三公”经费支出，让过“紧日子”成为政府工作常态。强化廉政廉洁教育，完善监督制约机制，使干部言有所戒、行有所止，按本色做人、按角色做事，持续提升创造力、战斗力、执行力、亲和力、抵抗力。

各位代表！

我们比任何时候都更加盼望明天的蚌埠，我们也将更加从容自信地面对蚌埠的明天！让我们紧密团结在以习近平同志为总书记的党中央周围，在省委、省政府和中共蚌埠市委的坚强领导下，创新进取，戮力同心，扎实苦干，向着全面建成小康社会、全面冲刺第一方阵的宏伟目标奋勇向前！
